# Risk factors of silent allograft fibrosis 10 years post-pediatric liver transplantation

**DOI:** 10.1038/s41598-020-58714-z

**Published:** 2020-02-04

**Authors:** Jinsoo Rhu, Sang Yun Ha, Sanghoon Lee, Jong Man Kim, Gyu-Seong Choi, Jae-Won Joh, Suk-Koo Lee

**Affiliations:** 1 Department of Surgery, Samsung Medical Center, Sungkyunkwan University School of Medicine, Seoul, Korea; 2 Department of Pathology, Samsung Medical Center, Sungkyunkwan University School of Medicine, Seoul, Korea

**Keywords:** Hepatocytes, Paediatric research

## Abstract

This study analyzed factors related to allograft fibrosis in clinically stable pediatric liver transplantation patients. Pediatric patients who underwent liver transplantation from January 1997 to January 2008 and further underwent 10-year protocol biopsies were examined. Grades of inflammation and fibrosis were classified based on Banff criteria and the Liver Allograft Scoring (LAF) system, respectively. Risk factors for fibrosis were analyzed using logistic regression. Sixty-six patients with no clinical signs of chronic liver disease were included. Forty-one patients out of 66 (62.1%) had certain stage of allograft fibrosis. More than five events with aminotransferase >50 U/L was a risk factor for a LAF score 1–2 portal fibrosis (OR = 3.156, CI 1.059–9.410, P = 0.039). More than five events with aminotransferase >100 U/L was a risk factor for LAF score 2 portal fibrosis (OR = 13.978, CI 2.025–97.460, P = 0.007) and LAF score 1–2 sinusoidal fibrosis (OR = 4.897, CI 1.167–20.548, P = 0.030). Positive autoantibody (OR = 3.298, CI 1.039–10.473, P = 0.043) and gamma-glutamyl transferase  60 U/L (OR = 6.201, CI 1.096–35.097, P = 0.039) were related to sinusoidal fibrosis with LAF score of 1–2 and 2, respectively. Experience of post-transplantation lymphoproliferative disease was related to LAF score 1–2 portal fibrosis (OR = 7.371, CI 1.320–41,170, P = 0.023) and LAF score 1–2 centrolobular fibrosis (OR = 8.822, CI = 1.378–56.455, P = 0.022). Our results indicate that liver fibrosis is common in patients with no clinical signs of graft deterioration and repeated elevation of aminotransferases, positive autoantibodies, elevated gamma-glutamyl transferase and experience of post-transplantation lymphoproliferative disease are suspicious signs for fibrosis.

## Introduction

Pediatric liver transplantation (LT) is now performed successfully in experienced transplantation centers, with a 10-year survival rate greater than 80%^1,2^. However, with the increase in long-term survival of pediatric LT patients, concerns are being raised about fibrotic changes in long-term allografts^3–5^. Risk factors for long-term allograft and biliary fibrosis were investigated by Scheenstra *et al*.^4^, who reported complications and cold ischemic time (CIT) as risk factors. Presence of positive autoantibodies with elevated immunoglobulin and *de novo* hepatitis C were found to be related to allograft fibrosis by Evans *et al*.^3^, while Venturi *et al*. noted correlation between portal fibrosis and prolonged ischemic time, deceased graft, and lymphoproliferative disease^5^. In their study, they also highlighted biliary complications as a related factor for sinusoidal fibrosis, while vascular complications, positive autoantibodies, and high gamma-globulin level were related to centrolobular fibrosis. Allograft fibrosis is particularly concerning in pediatric LT patients because these recipients have a longer life-expectancy than adult LT recipients. To avoid the need for re-transplantation, which is both surgically and immunologically challenging, maintaining the graft without fibrosis is vital. However, previous studies have reported relatively high rates of fibrosis between 20% and 30% based on microscopic examination^1,3–5^.

For the transplant clinician, allograft fibrosis on histology can be an unexpected finding if the patient had an uneventful course during follow up. Fibrosis results from the wound healing response to repeated stimuli. Our hypothesis in this study was that, even in patients with no clinical signs of chronic liver disease, repeated stimuli would result in notable changes in clinical factors. We designed this study to elucidate the factors related to allograft fibrosis that can act as markers for transplant clinicians to detect and limit slowly progressing liver fibrosis in clinically silent pediatric LT patients.

## Methods

### Patients and data

Information from pediatric patients who underwent LT at Samsung Medical Center in Seoul, Korea, between January 1997 and January 2008 was gathered from a prospectively maintained database and reviewed retrospectively. Recipients’ demographic, medical, transplantation, and follow-up data were collected. Medical data comprised etiology of liver disease, laboratory data, and pediatric end-stage liver disease (PELD) score at the time of LT. Data related to transplantation were ABO incompatibility, donor sex and age, graft type, macro- and microsteatosis of the graft liver, operation time, CIT, warm ischemic time (WIT), and performance of hepaticojejunostomy (HJ). Follow-up data included episodes of biopsy-proven acute rejection (BPAR) and complications and readmission as well as immunosuppressive regimen. Laboratory data comprised trough concentration of tacrolimus and liver function tests such as total bilirubin (TB), aspartate aminotransferase (AST), alanine aminotransferase (ALT), and gamma-glutamyl transferase (GGT). Tests for serum gamma-globulin (immunoglobulin G) level and autoantibodies were performed at the time of the 10-year protocol biopsy, and data regarding fluorescent antinuclear antibody (FANA), antimitochondrial antibody (AMA), and smooth muscle antibody (SMA) positivity were also collected.

Only LT recipients with a normally functioning liver without any symptoms of liver fibrosis/cirrhosis were included in the study. Inclusion criteria at the time of protocol biopsy were TB < 3.0 mg/dL. Recipients who were on the re-transplantation waiting list due to elevated liver function tests and signs of allograft fibrosis/cirrhosis such as ascites, varix formation, or growth retardation were excluded from the study.

### Immunosuppression

Patients were mainly on a double immunosuppressive regimen consisting of tacrolimus and a steroid. Mycophenolate mofetil was added for certain patients based on need for additional immunosuppression without increasing the dosage of tacrolimus. Tacrolimus was administered at an initial dose of 0.075 mg/kg, twice a day, and was titrated to a target trough concentration of 10 to 12 ng/mL during the first month after LT. Thereafter, it was lowered gradually to 8 to 10 ng/mL during the first to third months after LT and subsequently to 5 to 8 ng/mL, with further decreases based on the status of individual patients. The steroid was gradually tapered and terminated at around 3–6 months during the initial periods of our clinical practice. The protocol to terminate the steroid was shortened to around 1–2 months after LT since November 2001.

### Histological evaluation of the 10-year protocol biopsy

Patients underwent 10-year protocol biopsy around their 10-year post-LT mark. Our policy of performing protocol biopsies is based on initial studies that revealed inflammation and fibrosis even in patients with normal liver function tests. Evaluation of long-term histological changes in the allograft may promote long-term graft survival by allowing the medical team to perform interventions despite a normally functioning allograft.

Histological features were evaluated by an expert pathologist specializing in LT. Grade of inflammation was classified based on Banff criteria, while the stage of fibrosis was classified based on the liver allograft fibrosis (LAF) scoring system. Inflammation of the portal area, bile duct, and endothelium was scored, and the rejection activity index (RAI) was calculated. Portal, sinusoidal and centrolobular fibrosis were scored between 0 to 3.

### Statistical analysis

Mean tacrolimus trough concentration and mean TB level for the entire period from LT to the 10-year protocol biopsy were calculated for each individual using Microsoft Excel (Microsoft Corp., Redmond, WA, USA). Mean levels were calculated from laboratory tests performed after LT to the day of protocol biopsy, and the interval between laboratory tests was taken into account when calculating mean exposure.

After reviewing the clinical course of individual patients, the numbers of times aminotransferase level was above 50 U/L and above 100 U/L, respectively, were counted. Aminotransferase level was not evaluated the first month after LT because most recipients have elevated aminotransferase level during this period.

The significance of differences in grades of inflammation and portal fibrosis was assessed using various statistical methods as appropriate. Student’s t-test, one-way analysis of variance, the Kruskal–Wallis test, and the Mann–Whitney U test were used for continuous variables. For categorical variables, the chi-square test, Fisher’s exact test, or a linear-by-linear association test were used.

Separate analyses of risk factors for different scores of the component of LAF scoring system were performed using multivariable binary logistic regression analysis. Variables with a P-value < 0.05 in univariable analysis were included in multivariable analysis, which was performed using a backward likelihood ratio method.

All statistical analyses were performed using the Statistical Package for the Social Sciences version 20.0 (IBM Corp., Armonk, NY, USA). These data are available upon request.

### Ethical approval

This study was approved by the Institutional Review Board (IRB) of Samsung Medical Center (IRB no. 2018–11–090).

### Informed consent

The need for informed consent was waived by the IRB of Samsung Medical Center due to the retrospective nature of the study. Investigational methods used in this study were implemented in accordance with the relevant guidelines and regulations of the IRB.

## Results

**Figure 1:**
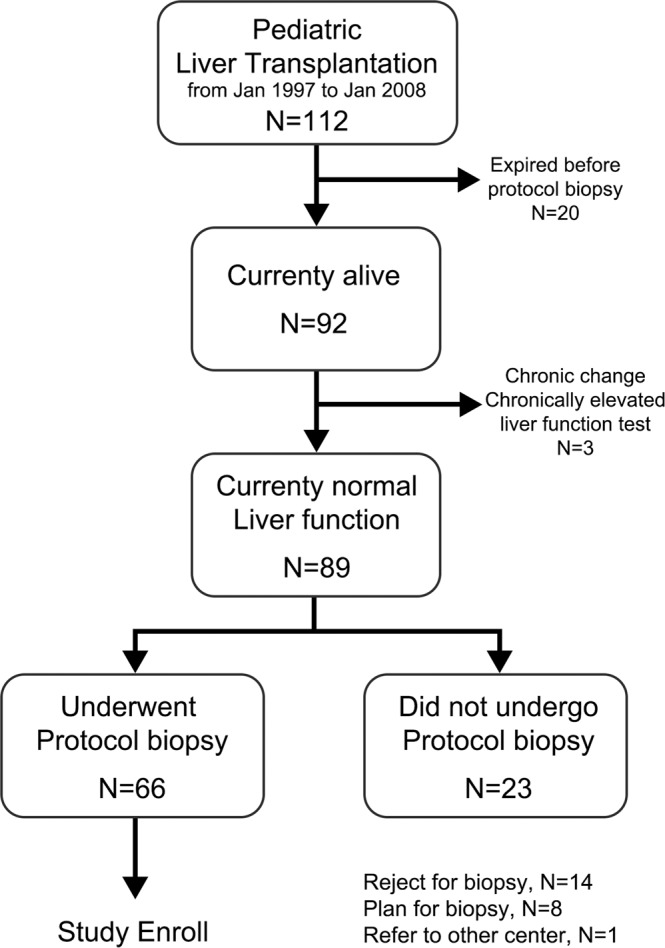
Flow diagram of patients included in the study. During the study period, 112 pediatric patients underwent LT at our center. After excluding 20 patients who died before undergoing 10-year protocol biopsy and three patients who had experienced chronic changes in elevated liver function tests, 66 of 89 patients with normal liver function tests who underwent protocol biopsy were included in the study.

During the study period, 112 patients underwent pediatric LT at Samsung Medical Center. Twenty patients died before the 10-year post-LT timepoint. Three patients were listed for re-transplantation for graft failure due to chronic graft dysfunction and already had elevated liver function test results before reaching the 10-year post-LT timepoint. Among the 89 patients with normal liver function tests, 23 did not undergo 10-year protocol biopsy for various reasons. Fourteen patients refused biopsy due to the invasive nature. Eight patients agreed to undergo a protocol biopsy but did not do so during the study period. One patient was referred to another transplantation center before reaching the 10-year mark for protocol biopsy. Therefore, a total of 66 patients (30 males and 36 females) was included in the present study (Fig. 1).

Baseline characteristics, data related to transplantation, and post-LT follow-up data as well as laboratory results at the time of 10-year post-LT biopsy are summarized in Table 1. Median recipient age at LT was 11.6 months, with an interquartile range (IQR) of 14.3 months. Mean donor age was 32.6 ± 6.2 years. Median PELD score was 13 (IQR: 12). The most common etiology was biliary atresia (n = 53; 80.3%). Most LTs were living-donor LTs (65/66; 98.5%), and the left lateral liver was most commonly used (n = 62/65; 93.9%). Mean biopsy interval was 134.7 ± 18.0 months. Additionally, mean trough FK concentration was 3.62 ± 1.06 ng/mL, and a mean number of 126.0 ± 43.6 tests for trough FK concentration were performed. Furthermore, the mean TB during the entire period was 0.67 ± 0.35 mg/dL, and 60 patients (90.9%) and 47 patients (71.2%) experienced elevation of aminotransferase level above 50 U/L and 100 U/L, respectively. Median number of times aminotransferase was elevated above 50 U/L was 4.5 (range: 0–18), while the median number of times aminotransferase was elevated above 100 U/L was 1.5 (range: 0–13). Median TB, AST, ALT, creatinine, and GGT values were 0.5 mg/dL (IQR: 0.5), 23.5 U/L (IQR: 12.5), 17.0 (IQR: 10.0), 0.53 mg/dL (IQR: 0.15), and 20.5 U/L (IQR: 27.0), respectively. Twenty-nine patients (43.9%) experienced acute allograft rejection during the 10-year post-LT period. Although there was no recipient with hepatitis B surface antigen positive at the time of LT, *de novo* hepatitis B occurred in three patients (4.5%) who were transplanted with hepatitis B core antibody positive donors.

**Table 1:** Baseline characteristic of clinically stable pediatric LT recipients who underwent 10-year protocol biopsy.

Factor	No. of patients	%
Recipient sex (M/F)	30/36	45.5/54.5
Recipient age, median (months, IQR)	11.6 (14.3)	
Donor sex (M/F)	41/25	62.1/37.9
Donor age, mean (years)	32.6 ± 6.2	
ABO unidentical	18	27.3
ABO incompatible	2	3.0
Donor anti-HBc antibody–positive	23	34.8
EBV high risk	42	63.6
Cytomegalovirus status		
Recipient IgG(+)/donor IgG (+)	53	80.3
Recipient IgG(−)/donor IgG (+)	13	19.7
Median PELD score (IQR)	13 (12)	
Etiology		
Biliary atresia	53	80.3
Neonatal hepatitis	2	3.0
Hepatoblastoma	2	3.0
Wilson’s disease	3	4.5
Alagile syndrome	1	1.5
Hemagioendothelioma	1	1.5
Fulminant hepatic failure	3	4.5
Glycogen storage disease	1	1.5
Living donor	65	98.5
Graft type		
Whole liver	1	1.5
Left lateral liver	62	93.9
Left hemiliver	1	1.5
Right hemiliver	2	3.0
Steatosis		
Macro, median (IQR, %)	5 (6)	
Micro, median (IQR, %)	5 (10)	
Operation time, median (minutes)	380 (142)	
CIT, median (minutes)	49 (55)	
WIT, median (minutes)	33.5 (13)	
Hepaticojejunostomy	58	87.9
Mean biopsy interval (months)	134.7 ± 18.0	
Mean trough FK level during entire period (ng/mL)	3.62 ± 1.06	
Mean number of FK trough level measurements (n)	126.0 ± 43.6	
Mean TB during entire period (mg/dL)	0.67 ± 0.35	
Episodes during post-LT period		
Elevation of AST/ALT above 50 U/L (n)	60	90.9
Median number of episodes per patient (n, range)	4.5 (0–18)	
Elevation of AST/ALT above 100 U/L (n)	47	71.2
Median number of episodes per patient (n, range)	1.5 (0–13)	
Laboratory results at the time of biopsy		
Median TB (mg/dL)	0.5 (0.5)	
Median AST (U/L)	23.5 (12.5)	
Median ALT (U/L)	17.0 (10.0)	
Median creatinine (mg/dL)	0.53 (0.15)	
Median GGT (U/L)	20.5 (27.0)	
Detection of autoantibodies		
FANA	22	33.3
AMA	3	4.5
SMA	4	6.1
Mean serum IgG	1195 ± 529	
Experience of acute rejection (n)	29	43.9
Median number of episodes (range)	0 (0–4)	
Median duration until the first episode (months)	3.26 (1.33–38.1)	
Experience of biliary complication	5	7.6
Experience of portal vein complication	5	7.6
Experience of hepatic vein complication	1	1.5
Experience of PTLD	9	13.6
De novo hepatitis B	3	4.5

Interquartile range IQR, Cold ischemic time CIT, Warm ischemic time WIT, Aspartate aminotransferase AST, Alanine aminotransferase ALT, Total bilirubin TB, Gamma glutamyl transferase GGT, Fluorescent antinuclear antibody FANA, Anti-mitochondrial antibody AMA, Smooth muscle antibody SMA, Post-transplantation lymphoproliferative disease PTLD.

*Normal values of the laboratory of Samsung Medical Center are 0~1.2 mg/dL for TB, 0~32 U/L for AST, 0~33 U/L for ALT, 6~42 U/L for GGT, and 700~1600 mg/dL for IgG.

Table 2 summarizes the histological features of the 10-year post-LT biopsies. Based on the Banff criteria for acute rejection, normal, indeterminate, mild, and moderate rejection were observed in 34 (51.5%), 23 (34.8%), eight (12.1%), and one (1.5%) patients, respectively. Based on LAF scoring system, patients with no fibrosis were 37.9% (n = 25) while the other patients had certain stage of fibrosis.

**Table 2:** Histologic findings regarding allograft inflammation and fibrosis classified based on Banff criteria for acute allograft rejection and liver allograft fibrosis scoring, respectively.

Factor	No. of patients	%
**Banff criteria for acute rejection**		
Normal, RAI = 0	34	51.5
Indeterminate, RAI = 1–2	23	34.8
Mild rejection, RAI = 3–4	8	12.1
Moderate rejection, RAI = 5–7	1	1.5
Severe rejection, RAI = 8–9	—	—
Portal inflammation score		
0	34	51.5
1	24	36.4
2	6	9.1
3	2	3.0
Bile duct damage score		
0	57	86.4
1	8	12.1
2	1	1.5
3	—	—
Venous endothelial inflammation score		
0	59	89.4
1	7	10.6
2	—	—
3	—	—
**LAF score for Fibrosis**		
Portal and periportal fibrosis		
0	41	62.1
1	18	27.3
2	7	10.6
3	—	—
Perisinusoidal		
0	46	69.7
1	13	19.7
2	7	10.6
3	—	—
Centrilobular		
0	47	71.2
1	17	25.8
2	2	3.0
3	—	—
Total		
0	25	37.9
1	20	30.3
2	9	13.6
3	6	9.1
4	6	9.1

Rejection activity index RAI, Liver allograft fibrosis LAF.

### Comparisons among grades of inflammation based on Banff criteria

Patient characteristics according to grades of inflammation as assessed by the Banff criteria for acute allograft rejection are presented in Table 3. All characteristics were similar among RAI 0 (n = 34), RAI 1 to 2 (n = 23), and RAI ≥ 3 (n = 9) groups; the only difference among RAI groups was rejection rate (29.4% in RAI 0, 60.9% in RAI 1 to 2, and 55.6% in RAI ≥ 3, P = 0.039). There was a significant difference in median ALT level at the time of protocol biopsy between RAI 0 and RAI 1 to 2 (P = 0.012). However, median value of ALT was within the normal range in all RAI groups.

**Table 3:** Comparisons of characteristics of pediatric LT recipients based on Banff criteria for acute rejection grade observed in 10-year post-transplantation protocol biopsy.

Variables	Banff criteria for acute rejection			P-value
	RAI 0 (n = 34)	RAI 1–2 (n = 23)	RAI ≥ 3 (n = 9)	
Recipient sex (Male, %)	12 (35.3%)	13 (56.5%)	5 (55.6%)	0.133
Median recipient age (months)	11.0 (13.4)	17.6 (14.5)	8.2 (107.5)	0.345
Donor sex (Male, %)	21 (61.8%)	14 (60.9%)	6 (66.7%)	0.851
Median donor age (years)	32.5 (5)	31.0 (7)	33.0 (11)	0.889
ABO non-identical (%)	8 (23.5%)	7 (30.4%)	3 (33.3%)	0.484
ABO incompatible (%)	1 (2.9%)	1 (4.3%)	—	0.808
Laboratory results at the time of LT				
Median TB (mg/dL)	16.2 (16.0)	14.3 (14.2)	9.2 (15.5)	0.736
Mean albumin (g/dL)	3.02 ± 0.70	3.08 ± 0.67	3.06 ± 0.61	0.938
Median PT/INR	1.36 (0.45)	1.29 (0.54)	1.30 (0.32)	0.845
Median serum creatinine (mg/dL)	0.42 (0.33)	0.44 (0.38)	0.45 (0.45)	0.873
Median PELD score	14 (15)	11 (14)	11.5 (10)	0.500
Median biopsy interval (months)	133.0 (15.0)	129.2 (14.7)	134.2 (18.4)	0.901
Mean trough FK of entire period, median (ng/mL)	3.25 (1.05)	3.47 (2.17)	3.19 (2.62)	0.235
Mean number of tests for FK, median	108 (32)	135 (59)	103 (57)	0.080
Mean TB of entire period, median (mg/dL)	0.61 (0.36)	0.55 (0.34)	0.53 (0.61)	0.736
Experience of rejection (n, %)	10 (29.4%)	14 (60.9%)	5 (55.6%)	0.039
Experience of biliary complication (n/%)	3 (8.8%)	2 (8.7%)	—	0.474
Experience of PTLD (n/%)	2 (5.9%)	6 (26.1%)	1 (11.1%)	0.229
Events with AST/ALT > 50 U/L, median	3 (7)	6 (8)	6 (7)	0.061
Events with AST/ALT > 100 U/L, median	1 (3)	2 (6)	2 (2)	0.320
Laboratory results at biopsy				
Median TB (mg/dL, IQR)	0.5 (0.6)	0.5 (0.6)	0.5 (0.6)	0.970
Median AST (U/L, IQR)	22 (12)	26 (24)	29 (30.5)	0.248
Median ALT (U/L, IQR)	15 (10)	21 (33)	20 (47)	0.042*
Median GGT (U/L, IQR)	20 (20.5)	24 (44)	26 (88)	0.382
Median creatinine (g/dL, IQR)	0.54 (0.12)	0.56 (0.27)	0.53 (0.67)	0.434
Median IgG level (mg/dL, IQR)	1036 (306)	1152 (466)	1185 (720)	0.596
Positive for FANA (n/%)	8 (23.5%)	10 (43.5%)	4 (44.4%)	0.115
Positive for AMA (n/%)	2 (5.9%)	—	1 (11.1%)	0.911
Positive for SMA (n/%)	2 (5.9%)	2 (8.7%)	—	0.728
Positive for any autoantibodies (n/%)	10 (29.4%)	11 (47.8%)	4 (44.4%)	0.220

Rejection activity index RAI, Interquartile range IQR, Liver transplantation LT, Total bilirubin TB, Prothrombin time/international normalized ratio PT/INR, Post-transplantation lymphoproliferative disease PTLD, Aspartate aminotransferase AST, Alanine aminotransferase ALT, Gamma glutamyl transferase GGT, Fluorescent antinuclear antibody FANA, Anti-mitochondrial antibody AMA, Smooth muscle antibody SMA.

*P = 0.012 by Mann-Whiteney test between RAI = 0 and RAI = 1–2 groups.

### Comparisons of stages of portal fibrosis based on LAF scoring system

Patient characteristics according to portal fibrosis based on LAF scoring system are compared in Table 4. Regarding portal fibrosis, median TB at the time of LT (P = 0.024), experience of rejection (P = 0.031), experience of PTLD (P = 0.015), median number of events with AST/ALT above 50 U/L (P = 0.010) and 100 U/L (P = 0.042) showed significant differences among different scores. Regarding, sinusoidal fibrosis, median donor age (P = 0.003), ABO incompatibility (P = 0.001), positive FANA (P = 0.007), and positivity rate for any autoantibodies (P = 0.011) were significantly different among different scores. Regarding centrolobular fibrosis, donor sex (P = 0.001), median donor age (P = 0.001), experience of PTLD (P = 0.002), median ALT (P = 0.011), median GGT (P = 0.023), and median creatinine (P = 0.039) at the time of biopsy were significantly different among patients with or without centrolobular fibrosis.

**Table 4:** Comparisons of characteristics of pediatric LT recipients based on LAF scoring for allograft fibrosis observed in 10-year post-transplantation protocol biopsy.

Variables	LAF score, portal				LAF score, sinusoidal				LAF score, centrilobular		
	0 (n = 41)	1 (n = 18)	2 (n = 7)	P	0 (n = 46)	1 (n = 13)	2 (n = 7)	P	0 (n = 47)	1–2 (n = 19)	P
Recipient sex (Male)	19 (46.3%)	6 (33.3%)	5 (71.4%)	0.600	20 (43.5%)	6 (46.2%)	4 (57.1%)	0.529	22 (46.8%)	25 (53.2%)	0.728
Median recipient age (months)	11.6 (16.0)	11.7 (17.4)	23.2 (74.3)	0.208	11.8 (25.4)	11.9 (12.6)	18.7 (37.6)	0.798	11.9 (30.9)	12.1 (11.6)	0.357
Donor sex (Male)	24 (58.5%)	11 (61.1%)	6 (85.7%)	0.247	30 (65.2%)	5 (38.5%)	6/1 (85.7%)	0.932	23 (48.9%)	18 (94.7%)	0.001
Median donor age (years)	33 (5)	30.5 (9)	32 (14)	0.595	33 (7)	30 (6)	34 (9)	0.003	34 (7)	30 (3)	0.001
ABO non-identical (%)	10 (24.4%)	7 (38.9%)	1 (14.3%)	0.912	12 (26.1%)	3 (23.1%)	3 (42.9%)	0.505	13 (27.7%)	5 (26.3%)	0.912
ABO incompatible (%)	1 (2.4%)	1 (5.6%)	—	0.975	—	—	2 (28.6%)	0.001	1 (2.1%)	1 (5.3%)	0.496
Median graft weight (g)	261 (89.7)	266.5 (116)	283 (178)	0.609	270 (98.5)	257 (61.0)	282 (311.5)	0.284	269 (159.5)	264.5 (72.8)	0.664
Median graft-recipient weight ratio (%)	2.81 (1.33)	3.50 (1.39)	1.79 (1.09)	0.084	3.00 (1.58)	2.67 (1.23)	2.74 (2.42)	0.862	2.72 (1.82)	2.81 (1.11)	0.431
Laboratory results at LT											
Median TB (mg/dL)	16.2 (15.7)	15.4 (10.6)	3.1 (12.1)	0.024	13.3 (16.0)	17.3 (17.1)	14.3 (10.3)	0.957	13.7 (13.7)	16.1 (20.5)	0.972
Mean albumin (g/dL)	3.04 ± 0.67	2.96 ± 0.79	3.27 ± 0.75	0.589	3.01 ± 0.70	3.11 ± 0.49	3.16 ± 0.79	0.811	3.03 ± 0.71	3.07 ± 0.58	0.830
Median PT/INR	1.37 (0.44)	1.34 (0.61)	1.19 (0.22)	0.226	1.31 (0.45)	1.42 (0.50)	1.41 (0.45)	0.966	1.37 (0.44)	1.32 (0.45)	0.729
Median serum creatinine (mg/dL)	0.43 (0.35)	0.31 (0.44)	0.44 (0.31)	0.731	0.45 (0.33)	0.31 (0.32)	0.43 (0.53)	0.689	0.45 (0.35)	0.31 (0.40)	0.465
Median PELD score	14.5 (13)	10.5 (12)	5.0 (10)	0.053	14 (14)	14 (13)	11 (15)	0.976	14 (13)	11 (15)	0.642
Median biopsy interval (months)	132 (16.1)	132 (16.2)	136 (17.2)	0.917	134 (12.4)	129 (17.5)	132 (16.0)	0.727	134 (12.8)	127 (21.4)	0.243
Mean FK trough concentration, median (ng/mL)	3.16 (0.95)	3.70 (1.69)	5.16 (2.83)	0.088	3.39 (1.01)	4.03 (2.76)	3.13 (1.64)	0.834	3.47 (1.59)	3.12 (0.77)	0.131
Median number of tests for FK	114 (41)	112.5 (63)	146 (106)	0.175	111 (40)	123 (97)	156 (71)	0.333	115 (51)	116 (48)	0.750
Mean TB of entire period, median (mg/dL)	0.52 (0.33)	0.58 (0.33)	0.82 (0.67)	0.123	0.55 (0.34)	0.60 (0.55)	0.55 (0.48)	0.800	0.64 (0.32)	0.53 (0.38)	0.154
Experience of rejection (n, %)	15 (36.6%)	8 (44.4%)	6 (85.7%)	0.031	18 (39.1%)	6 (46.2%)	5 (71.4%)	0.131	20 (42.6%)	9 (47.4%)	0.721
Experience of vascular complication (n, %)	4 (9.8%)	2 (11.1%)	—	0.570	4 (8.7%)	2 (15.4%)	—	0.774	5 (10.6%)	1 (5.3%)	0.664
Experience of biliary complication (n, %)	4 (9.8%)	—	1 (14.3%)	0.773	4 (8.7%)	—	1 (14.3%)	0.975	3 (6.4%)	2 (10.5%)	0.621
Experience of PTLD (n, %)	2 (4.9%)	5 (27.8%)	2 (28.6%)	0.015	4 (8.7%)	3 (23.1%)	2 (28.6%)	0.080	2 (4.3%)	7 (36.8%)	0.002
Events with AST/ALT > 50 U/L, median	3 (7)	4.5 (8)	12 (8)	0.010	3 (7)	7 (13)	5 (10)	0.426	5 (8)	3 (8)	0.279
Events with AST/ALT > 100 U/L, median	1 (3)	1 (2)	7 (5)	0.042	1 (3)	2 (7)	3 (6)	0.154	2 (3)	1 (4)	0.535
Laboratory results at biopsy											
Median TB (mg/dL)	0.5 (0.35)	0.5 (0.53)	0.6 (0.9)	0.554	0.5 (0.35)	0.5 (0.65)	0.5 (0.7)	0.628	0.5 (0.5)	0.4 (0.6)	0.475
Median AST (U/L)	22 (11.5)	24 (12.3)	42 (27)	0.484	22 (11.3)	24 (23)	32 (21)	0.145	24 (14)	22 (11)	0.383
Median ALT (U/L)	16 (10)	18.5 (11.5)	48 (35.0)	0.079	18 (8.5)	16 (26.5)	19 (34)	0.739	21 (14)	14 (9)	0.011
Median GGT (U/L)	22 (26.5)	22.5 (39.8)	39.0 (169)	0.632	21.5 (19.5)	24.0 (42.0)	60.0 (82.0)	0.267	25 (43)	19 (11)	0.023
Median creatinine (g/dL)	0.52 (0.13)	0.55 (0.32)	0.63 (0.40)	0.273	0.54 (0.20)	0.49 (0.26)	0.56 (0.31)	0.751	0.54 (0.31)	0.49 (0.18)	0.039
Median IgG level (mg/dL)	1038 (360)	1185 (447)	1143 (543)	0.263	1058 (326)	1198 (619)	1020 (489)	0.851	1137 (343)	1020 (345)	0.144
Positive for FANA (n/%)	10 (24.4%)	9 (50.0%)	3 (42.9%)	0.098	11 (23.9%)	6 (46.2%)	5 (71.4%)	0.007	14 (29.8%)	8 (42.1%)	0.551
Positive for AMA (n/%)	3 (7.3%)	—	3 (42.9%)	0.209	2 (4.3%)	—	1 (14.3%)	0.501	3 (6.4%)	—	1.000
Positive for SMA (n/%)	2 (4.9%)	2 (11.1%)	—	0.964	3 (6.5%)	1 (7.7%)	—	0.629	3 (6.4%)	1 (5.3%)	0.393
Positive for any autoantibodies (n/%)	12 (29.3%)	10 (55.6%)	—	0.151	13 (28.3%)	7 (53.8%)	5 (71.4%)	0.011	17 (36.2%)	8 (42.1%)	0.653

*Rejection activity index RAI, Interquartile range IQR, Liver transplantation LT, Total bilirubin TB, Prothrombin time/international normalized ratio PT/INR, Post-transplantation lymphoproliferative disease PTLD, Aspartate aminotransferase AST, Alanine aminotransferase ALT, Gamma glutamyl transferase GGT, Fluorescent antinuclear antibody FANA, Anti-mitochondrial antibody AMA, Smooth muscle antibody SMA.

### Risk factors for different scores of fibrosis categorized by LAF scoring at 10 years

To analyze risk factors related to allograft fibrosis, multivariable logistic regression analyses of variables with a P-value < 0.05 in univariable analyses were performed (Table 5).

**Table 5:** Multivariable logistic regression analyses on potential risk factors for different scores of LAF scoring system.

LAF score	Variables	No.	OR	95% CI	P-value
Portal 1–2	AST/ALT above 50 U/L > 5	28	3.156	1.059–9.410	0.039
	Experience of PTLD	9	7.371	1.320–41.170	0.023
Portal 2	AST/ALT above 100 U/L > 5	11	13.978	2.025–96.460	0.007
	Experience of rejection	29	4.261	0.399–45.448	0.230
Sinusoidal 1–2	AST/ALT above 100 U/L > 5	11	4.897	1.167–20.548	0.030
	At the time of protocol biopsy				
	IgG ≥ 1,700 mg/dL	5	3.414	0.262–44.461	0.349
	GGT ≥ 50 U/L	15	2.832	0.711-	0.140
	Positive for any autoantibody	25	3.298	11.278 1.039–10.473	0.043
Sinusoidal 2	At the time of protocol biopsy				
	GGT ≥ 60 U/L	14	6.201	1.096–35.097	0.039
	Positive for FANA	22	5.880	0.963–35.904	0.055
Centrilobular 1–2	Donor sex (Female against Male)	25	0.021	0.001–0.345	0.007
	Donor age ≥ 30 years	50	0.049	0.005–0.460	0.008
	Experience of PTLD	9	8.822	1.378–56.455	0.022

Liver allograft fibrosis LAF, aspartate aminotransferase AST, alanine aminotransferase ALT, post-transplantation lymphoproliferative disease PTLD, gamma-glutamyl transferase GGT, Fluorescent antinuclear antibody FANA.

*Presented variables were significant factors analyzed in the univariable analyses.

Regarding LAF score 1–2 for portal fibrosis, more than five events of AST/ALT above 50 U/L (OR = 3.156, CI = 1.059–9.410, P = 0.039) and experience of PTLD (OR = 7.371, CI = 1.320–41.170, P = 0.023) were significant factors. On the other hand, more than five events of AST/ALT above 100 U/L (OR = 13.978, CI = 2.025–96.460, P = 0.007) was the only significant factor related to LAF score 2 for portal fibrosis while experience of rejection (OR = 4.261, CI = 0.399–45.448, P = 0.230) was only significant in the univariable anlaysis.

Regarding LAF score 1–2 for sinusoidal fibrosis, more than five events of AST/ALT above 50 U/L (OR = 4.897, CI = 1.167–20.548, P = 0.030) and positive for any autoantibodies (OR = 3.298, CI = 1.039–10.473, P = 0.043) were significant factors. IgG ≥ 1,700 mg/dL (OR = 3.414, CI = 0.262–44.461, P = 0.349) and GGT ≥ 50 U/L (OR = 2.832, CI = 0.711–11.278, P = 0.140) were only significant in the univariable analysis. On the other hand, GGT ≥ 60 U/L (OR = 6.201, CI = 1.096–35.097, P = 0.039) showed significant relationship to LAF score 2 for sinusoidal fibrosis while positive FANA (OR = 5.880, CI = 0.963–35.904, P = 0.055) showed trend toward significance.

Regarding centrolobular fibrosis, patients who were donated from female donor (OR = 0.021, CI = 0.001–0.345, P = 0.007) and from donor age ≥ 30 years (OR = 0.049, CI = 0.005–0.460, P = 0.008) showed decreased risk. Experience of PTLD showed significantly increased risk of LAF score 1–2 centrolobular fibrosis (OR = 8.822, CI = 1.378–56.455, P = 0.022).

## Discussion

We analyzed relationships between clinical parameters and liver allograft fibrosis using LAF scoring system at the 10-year post-LT follow-up visit in pediatric LT patients. While many patients had relatively normal liver function test results at the time of biopsy, allograft fibrosis was present in almost two third (62.1%) of the patients. Since our institute has started to perform 10-year post-LT protocol biopsy for pediatric LT patients, we have encountered patients with allograft fibrosis on histology despite a clinically well-behaving liver according to laboratory tests and radiologic imaging. We designed the present study to investigate potential risk factors related to development of fibrosis in pediatric LT patients and to elucidate potential cause(s) based on clinical course.

The first study to report the prevalence and clinical course of chronic hepatitis in pediatric LT recipients included 158 asymptomatic recipients who were evaluated by biopsy 1, 5, and 10 years post-LT^3^. The most common finding on histology was chronic hepatitis, with a prevalence of 22%, 43%, and 64% at 1, 5, and 10 years post-LT, respectively^3^. In the same study, the incidence of fibrosis associated with chronic hepatitis increased from 52% (13/25) in the first year to 91% (37/41) 10 years post-LT. Other studies have reported a similar high prevalence of graft inflammation ranging from 22% to 74% and graft fibrosis ranging from 27% to 97% in liver biopsies obtained more than 1 year post-LT^6–12^. Some studies reported that inflammation and fibrosis may improve with increased immunosuppression^10,13^.

Graft fibrosis in the absence of evident graft hepatitis has also been reported in several studies^4,14,15^. These studies claimed that fibrosis was not related to rejection, chronic hepatitis, or immunosuppressants^4,16^. Rather, fibrosis showed association with CIT and reduced-size grafts. The more severe was the fibrosis, the higher was the cholestatic liver chemistry test result.

We found that nearly two third of the patients evaluated (n = 41/66; 62.1%) had some stages of portal, sinusoidal or centrolobular fibrosis based on LAF scoring system. Although no patient had LAF score of 3 for the three components of LAF scoring system, LAF score 2 for portal, sinusoidal and centrolobular fibrosis were present in 7 (10.6%), 7 (10.6%) and 2 (3.0%) patients, respectively. Based on our inclusion of patients with a clinically well-behaving liver, the fibrosis rate of 62.1% is quite surprising high.

When we compared clinical variables among patients with different stages of fibrosis from different histological location, we found that different location of fibrosis showed different clinically related features. Portal fibrosis both LAF score of 1–2 and 2 showed relationship with events of elevated AST/ALT. Patients with more than five events of AST/ALT > 50 U/L was related to LAF score 1–2 portal fibrosis while patients with more than five events of AST/ALT > 100 U/L was related to LAF score 2 portal fibrosis. These findings show that histologically unevaluated events of elevated liver function tests can be related to allograft fibrosis. More than five events of AST/ALT > 100 U/L were also related to mild stage of sinusoidal fibrosis. Sinusoidal fibrosis also showed relationship with positive autoantibodies. Patients with positivity for any autoantibodies were significantly related to LAF score 1–2 sinusoidal fibrosis. Although statistically insignificant, positive for FANA showed a trend toward significance in the multivariable analysis for LAF score 2 sinusoidal fibrosis. Elevated GGT ≥ 60 U/L was the only significant factor related to LAF score 2 sinusoidal fibrosis. Elevated GGT ≥ 50 U/L was only significant in the univariable analysis for LAF score 1–2 sinusoidal fibrosis. These findings show that sinusoidal fibrosis can be related to the process of autoimmunity against liver graft whether or not of the presence of autoimmune hepatitis and also can show elevated GGT and histories of elevation of liver function tests. Centrolobular fibrosis showed relationship to the donor factor including sex and age. Female donor and age ≥ 30 years showed significantly decreased risk of centrolobular fibrosis with LAF score of 1–2. Experience of PTLD was related to LAF score 1–2 portal fibrosis and centrolobular fibrosis. Most of the factors that were significantly related to different location of fibrosis were previously reported by other studies. However, relationship of centrolobular fibrosis with the donor sex and donor age are new findings of our study. Unfortunately, explanation of these finding can be limited since we did not perform a protocol for a serial biopsy that can examine the change in the allograft.

Another interesting finding of our study is that there was no significant difference in clinical parameters according to grade of inflammation based on Banff criteria for acute allograft rejection, with the exception of a previous experience of rejection. Although inflammatory infiltrates are continuously present in allografts, only patients who have already experienced rejection are likely to experience repeat rejection. However, other variables, especially laboratory data at the time of biopsy, did not differ between inflammation groups. Furthermore, laboratory values were all within normal ranges.

Our findings have the important clinical implication that, despite normal liver function without clinical symptoms, there may be ongoing fibrosis occurring inside the allograft or rejection of the allograft. Most pediatric LT patients in this study underwent transplantation during their first year of life, and it is important that these patients maintain a healthy allograft due to their long life expectancy.

The management after the discovery of allograft fibrosis in recipients with normal laboratory values should also be proposed by the transplant clinicians. In our center, we increased the amount of immunosuppression by either increasing the dosage and trough level of calcineurin inhibitor or adding mycophenolic mofetil to the maintenance regimen. Those managements were based on the clinical courses of the recipients as well as the histological findings. However, whether these interventions were effective needs more evidence and further researches are required for the topic.

Since this study was designed as a retrospective study, there are several limitations that should be considered. Only 66 of a total of 112 pediatric LT patients (58.9%) underwent protocol biopsy during the study period, limiting the generalizability of the results. Another limitation was that we only examined 10-year post-LT histology. Additional biopsies other than the protocol biopsy were performed only in patients suspected to of rejection or hepatitis due to other causes. Furthermore, because our center has a protocol to taper steroid use early during post-LT management, we were not able to analyze the impact of steroids on allograft fibrosis. Previous studies have reported that steroid-free patients demonstrated increased fibrosis during the first year of the post-LT period^5^. However, our study analyzed the period 10 years post-LT, and continuing steroids for this length of time is not feasible.

In pediatric LT recipients, the possibility of autoimmune hepatitis also needs to be considered, even in recipients with normal liver function tests. Previous studies showed that positivity for serologic markers of autoimmune hepatitis is a risk factor for allograft fibrosis^3^. In our study, positive autoantibody was related to sinusoidal fibrosis. Two recipients were diagnosed with autoimmune hepatitis and had lymphoplasmacytic infiltration in the portal tract with interface activities. These two patients both had normal liver function test results. Stage of fibrosis was 1 for portal fibrosis and 1 for sinusoidal fibrosis in one patient and 2 for portal fibrosis and 1 for sinusoidal fibrosis in the other patient. Both had positive FANA with IgG level of 1957 and 4685 mg/dL, respectively. While only two patients had autoimmune hepatitis, their histological findings showing portal and sinusoidal fibrosis suggests the importance of autoimmunity of liver allograft upon liver allograft fibrosis in combination with the positive relationship of autoantibodies with sinusoidal fibrosis.

In our study population, three patients who were initially negative for hepatitis B surface antigen experienced subsequent *de novo* hepatitis B after receiving liver from hepatitis B core antibody positive donors. Though they showed relatively normal liver function test results during follow-up, two of these three patients had LAF score ≥2 based on the 10-year post-LT protocol biopsy. Although *de novo* hepatitis B was not a significant risk factor in the multivariable analysis, adequate control of viral hepatitis is vital in maintaining an allograft. No patients in our study population had viral hepatitis C.

Although there are still several questions that should be answered to better understand long-term graft function in pediatric LT patients, we discovered two parameters that may reflect the presence of allograft fibrosis. We found that recurrent elevation of AST/ALT levels may be a risk factor for portal and sinusoidal fibrosis. Positive autoantibodies and GGT at the time of biopsy shows the possibility of sinusoidal fibrosis. PTLD can also be related to portal and centrolobular fibrosis. Although more explanation is required, liver graft from male donor and younger donor can be risk factor centrolobular fibrosis. It is important to note that patients who have at least one of the factors mentioned above can have mild allograft fibrosis even when suspicious clinical symptoms for liver fibrosis are absent.
